# Is sleep bruxism associated with smartphone use, neck pain, and sleep features among adolescents?

**DOI:** 10.1590/1807-3107bor-2025.vol39.010

**Published:** 2025-02-03

**Authors:** Ivana Meyer PRADO, Letícia Fernanda MOREIRA-SANTOS, Gabriela de Faria e Barboza HOFFMAM, Lucas Guimarães ABREU, Sheyla Márcia AUAD, Isabela Almeida PORDEUS, Júnia Maria SERRA-NEGRA

**Affiliations:** (a) Universidade Federal de Minas Gerais - UFMG, School of Dentistry, Department of Pediatric Dentistry, Belo Horizonte, Minas Gerais, Brazil.

**Keywords:** Sleep Bruxism, Smartphone, Neck Pain, Adolescent

## Abstract

An association between bruxism and musculoskeletal disorders, such as neck pain, has been established. This study investigated the association of possible sleep bruxism (PSB) activities, including grinding, bracing, and thrusting, with smartphone use, smartphone-induced neck pain, and sleep features. This cross-sectional study involved 403 Brazilian adolescents aged 11 to 19 years. A self-administered questionnaire was used to assess the severity of PSB, smartphone use, smartphone-induced neck pain, and sleep features (sleep duration and quality and history of nightmares). Sociodemographic factors, as well as snoring and drooling on the pillow, were considered potential confounders and were assessed based on reports from parents/caregivers. Participants were selected using multiple-stage sampling. Descriptive analysis and multinomial regression were performed (p ≤ 0.05). Adolescents reporting nightmares at least once a month (OR = 3.402; 95%CI: 1.315–8.802) and sometimes experiencing smartphone-induced neck pain (OR: 3.697, 95%CI: 1.103–12.388) were more likely to report moderate/severe grinding. Drooling on the pillow (OR = 3.105, 95%CI: 1.316–7.329), poor/fairly good sleep quality (OR = 2.717, 95%CI: 1.279–5.770), and smartphone-induced neck pain (OR = 3.227, 95%CI: 1.121–9.285) were associated with mild bracing. Adolescents experiencing nightmares once a week (OR = 3.209, 95% CI: 1.202– 8.565) had a higher prevalence of mild thrusting. Self-reported smartphone-induced neck pain, nightmares, poor/fairly good sleep quality, and drooling on the pillow were associated with a higher prevalence of PSB activities among Brazilian adolescents. Clinicians and researchers are encouraged to include assessments of smartphone use and sleep features in anamnesis, promoting a comprehensive approach to PSB, from diagnosis to treatment.

## Introduction

Sleep bruxism (SB) is a masticatory muscle activity during sleep, categorized as either rhythmic (phasic) or non-rhythmic (tonic).^1^Its etiology is multifactorial, encompassing psychological (e.g., stress, personality traits, and anxiety), biological (e.g., genetics, sleep arousal, and sleep-disordered breathing), and exogenous factors (e.g., smoking, alcohol, and caffeine).^2^The prevalence of SB among adolescents ranges from 9.2 to 16.9%.^3,4^ Bruxism activities involve clenching or grinding of the teeth and/or bracing or thrusting of the mandible, not necessarily involving tooth contact.^1^Based on its evaluation, SB can be graded as possible sleep bruxism (PSB), based on a positive self-report; probable sleep bruxism, based on a positive clinical inspection, with or without a positive self-report; or definite bruxism, based on a positive instrumental assessment, despite a positive self-report and/or a positive clinical inspection.^1^

Smartphones are universally accepted and used in modern society not only as cell phones but also as computers, media players, and video cameras, providing information at any time or place.^5^Excessive use of smartphones may negatively affect an individual’s sleep (e.g., quality and duration), circadian physiology, and the musculoskeletal system.^5-7^ Individuals usually exhibit a more forward-head posture when looking at their smartphone screens,^8^especially while texting.^5^The surge in smartphone use and addiction has been linked to the growing prevalence of neck pain, especially among adolescents.^9^

Smartphone use can influence an individual’s sleep, muscle activity, and head posture.^8^ SB has also been associated with sleep problems, muscle pain, and forward head posture.^10-12^Thus, it could be hypothesized that excessive smartphone use, especially at night, may influence SB activities in two ways. First, excessive smartphone use can trigger SB-related sleep problems. Second, smartphone-induced neck pain may be related to SB or even intensify its severity.^10^ Considering this background and the lack of data on the stomatognathic system and musculoskeletal disorders,^10^ this study aimed to evaluate the association of the severity of PSB activities (grinding, bracing, and thrusting) with smartphone use, smartphone-induced neck pain, and sleep features among Brazilian adolescents.

## Methods

### Study design, setting, and participants

This population-based cross-sectional study was conducted on adolescents enrolled in public and private schools from Belo Horizonte, a city located in southeastern Brazil, from August to December 2018. The city is organized into nine administrative regions. Participants were randomly selected using multiple-stage sampling. One private and one public school from each of the nine regions were randomly selected, resulting in 18 selected schools, all of which agreed to take part in the study. Subsequently, one classroom from each of the participating schools was randomly selected, and all students from those classrooms were invited to participate.

Inclusion criteria encompassed adolescents aged 11 to 19 years, enrolled in private and public schools. Exclusion criteria were applied to adolescents on antidepressant and/or anticonvulsant medications,^13^ as well as those with syndromes and/or cognitive disorders.^4^Participants who did not fully complete all assessment instruments were also excluded from the study. Evaluation of adolescents’ health status and medication use relied on reports provided by their parents/caregivers.

### Ethical aspects

This study was approved by the Research Ethics Committee of the Federal University of Minas Gerais (process #91561018.5.0000.5149) and was conducted in compliance with the Declaration of Helsinki. Adolescents and their parents/caregivers were informed about the study’s aims, assured of confidentiality, and informed of the voluntary nature of their participation through a consent form.

### Data collection

The instruments used for data collection were self-administered questionnaires answered by parents/caregivers and adolescents. Researchers visited the selected schools and distributed the consent forms and questionnaires for parents/caregivers of the adolescents, who took them home for their parents/caregivers to sign and complete. One week later, the researchers returned to the schools to collect the signed forms and questionnaires. Additionally, the researchers administered the questionnaires to adolescents at the school, contingent upon parental or caregiver consent.

### Parents or caregivers questionnaire

Parents/caregivers completed a questionnaire consisting of nine questions that addressed the family’s socioeconomic characteristics (e.g., relationship to the adolescent, age, sex, educational level, and monthly family income), the adolescent’s sleep features (e.g., history of drooling on the pillow and snoring), and the adolescent’s medical history, including medication use and/or reports of syndromes and/or cognitive disorders. Monthly family income was calculated as the sum of the number of Brazilian minimum wages (BMWs) of all family members. At the time of data collection, one BMW was equivalent to USD 283.77, and the variable was categorized as ≤ 3 BMW and > 3 BMW. Questions about the adolescents’ history of drooling on the pillow and snoring could be answered with “no,” “sometimes,” or “often.”

### Adolescent questionnaire

Adolescents self-completed a 20-item questionnaire at school, consisting of three questions about their sleep features (e.g., sleep duration, sleep quality, history of nightmares),^4^ three about the history of PSB activity (e.g., grinding, bracing, and thrusting),^1,4^ 13 questions related to smartphone use, and one about smartphone-induced neck pain.

The sleep features assessed included sleep duration, sleep quality, and history of nightmares, with all questions referring to the previous two weeks. Sleep duration was calculated based on the adolescent’s reported bedtime and wake-up time. Responses to the sleep quality questions were categorized as “poor/fairly good” and “good.” The question about the history of nightmares could be answered with “never/rarely,” “once a month,” or “once a week.”

PSB activities such as grinding, bracing, and thrusting of the mandible were evaluated by asking the following questions:^1^

- Sleep bruxism grinding activity item: Are you aware or has anyone told you that you have ground your teeth during sleep in the past two weeks?
- Sleep bruxism thrusting activity item: When awakening in the morning or waking up at night, over the past two weeks, was your jaw positioned forward or sideways?
- Sleep bruxism bracing activity item: When awakening in the morning or waking up at night, over the past two weeks, was your jaw in a steady/rigid position (making it difficult to open your mouth)?

The answer options for these questions were “no,” “sometimes,” or “often.” Grinding, bracing, and thrusting activities were categorized as “absent” if the activity did not occur in the past two weeks, “mild” if the activity occurred sometimes, and “moderate/severe” if the activity occurred often.^14^

The questions about smartphone use were based on the studies by Bartel et al.^15^ and Bruni et al.^16^ and they were as follows:

- Do you use or have access to a smartphone?
- Do you use your smartphone in bed before bedtime?
- Do you play games on your smartphone before bedtime?
- When you wake up in the morning, do you use your smartphone before doing anything else?
- Do you bring your smartphone to your bedroom when going to sleep?
- Do you use your smartphone in bed after turning the lights off to sleep?
- Do you think that you spend time that should be spent sleeping instead of using your smartphone?
- If you wake up in the middle of the night, do you use your smartphone?
- Think about the time you spend on your smartphone daily. On average, for how many hours do you use your smartphone daily?
- How old were you when you started using a smartphone?

Questions A, B, C, and G could be answered with “yes” or “no.” Questions D, E, F, and H could be answered with “no,” “sometimes,” or “often.” Questions I and J were open-ended questions regarding the number of hours and years, respectively.

The question about smartphone-induced neck pain was “Have you ever felt pain in your neck due to smartphone use?”. The answer options were “no,” “sometimes,” or “often.”

### Sample size calculation

The sample size was calculated based on the following parameters: a 95% confidence interval, a 4% standard error, and a 15.3% prevalence of PSB.^17^A minimum sample size of 312 adolescents was estimated. A correction factor of 1.2 was added to enhance study precision due to multiple-stage sampling, resulting in a minimum sample size requirement of 375 adolescents. Subsequently, the sample was increased by 20% to account for possible losses. The final sample included 449 adolescents.

### Pilot study

A pilot study was performed with 43 adolescents aged 11 to 19 years, approximately 10% of the final sample, at a public school. The pilot study aimed to ensure that adolescents fully understood the questionnaire prepared by the researchers and to test the proposed methodology. Throughout the administration of the questionnaire, the researchers remained on hand to clarify any questions the adolescents could have. Participants in the pilot study were not included in the main study sample. The pilot study indicated that the adolescents understood all questions, and as a result, no changes were made to the methodology.

### Statistical analysis

Data were analyzed using the SPSS program for Windows (version 21.0, SPSS Inc., Chicago, USA).^18^ Descriptive statistics (frequency, distribution, mean, and standard deviation) and multinomial logistical regression (adjusted and unadjusted models) were performed to evaluate the association between the severity of PSB activity (dependent variable) and sociodemographic characteristics, sleep features, smartphone use habits, and smartphone-induced neck pain (independent variables). All independent variables with a p-value ≤ 0.20 in the unadjusted analysis were included in the adjusted model. The significant level was set at 5.0%.

## Results

A total of 403 adolescents were included in the study, resulting in a high response rate of 92.7%. Thirty-two participants (7.3%) were excluded because of missing data. Most of the questionnaires were answered by mothers (81.5%), followed by fathers (14.0%) and others (4.5%), and the mean age of the respondents was 43.5 years (± 7.7). Most respondents reported a family income of three BMWs or less (60.8%). The mean age of the adolescents was 14.3 years (± 1.5), ranging from 11 to 19 years (median = 14 years). Females accounted for 58.1% of the sample, and 52.9% of them were enrolled in public schools.

Table 1 presents the descriptive analysis. Adolescents reported an average nightly sleep duration of 7.8 hours (± 1.4), and 34.7% (140) indicated they had poor/fairly good sleep quality. Mild grinding activity was observed in 16.1% of the adolescents, while mild thrusting and bracing activities were identified in 10.9% and 10.7%, respectively. All adolescents (100%) reported using/having access to a smartphone. Participants reported using their smartphones for an average of 8 hours (± 5.4) per day. Most adolescents (86.1%) reported using their smartphones in bed before bedtime and upon waking up (sometimes = 45.4%; often = 37.2%). Additionally, 77.9% often took their smartphones to their bedrooms, and 55.6% often used them in the dark after going to bed. Smartphone-induced neck pain was often reported by 14.6% of the adolescents and sometimes by 50.6%.

**Table 1:** Descriptive statistics of sleep features, possible sleep bruxism activity, smartphone use, and neck pain among Brazilian adolescents.

Variables	Frequency (%)
Snores during sleep	
No	240 (60.4)
Sometimes	138 (34.8)
Often	19 (04.8)
Drools on the pillow during sleep	
No	192 (48.0)
Sometimes	167 (41.8)
Often	41 (10.2)
Sleep duration	
Mean [SD]	07.8 [± 1.4]
Median [Min – Max]	07.6 [04 – 13.5]
Self-reported sleep quality	
Good	263 (65.3)
Poor/fairly good	140 (34.7)
Has nightmares	
Never/rarely	309 (76.7)
Once a month	61 (15.1)
Once a week	33 (08.2)
Possible sleep bruxism – bracing activity	
Absent	353 (87.6)
Mild	43 (10.7)
Moderate/severe	07 (01.7)
Possible sleep bruxism – grinding activity	
Absent	313 (77.7)
Mild	65 (16.1)
Moderate/severe	25 (06.2)
Possible sleep bruxism – thrusting activity	
Absent	345 (85.6)
Mild	44 (10.9)
Moderate/severe	14 (03.5)
Uses the smartphone in bed before bedtime	
No	54 (13.4)
Yes	347 (86.1)
Uses smartphone to play games before bedtime	
No	93 (23.1)
Yes	310 (76.9)
Uses smartphone upon waking up, before any other activity	
Never	70 (17.4)
Sometimes	183 (45.4)
Often	150 (37.2)
Takes the smartphone to the bedroom when going to sleep	
Never	31 (07.7)
Sometimes	58 (14.4)
Often	314 (77.9)
Uses the smartphone with lights off after going to sleep	
Never	63 (15.6)
Sometimes	116 (28.8)
Often	224 (55.6)
Thinks that spends time that should be spent sleeping instead of using the smartphone	
No	237 (58.8)
Yes	166 (41.2)
Uses the smartphone when waking up in the middle of the night	
Never	211 (52.4)
Sometimes	155 (38.5)
Often	37 (09.2)
Hours the adolescent thinks they spend on their smartphone per day	
Mean [SD]	08.0 [± 05.4]
Median [Min – Max]	07.0 [0 – 24]
Age when first started using the smartphone	
Mean [SD]	10.0 [± 02.1]
Median [Min – Max]	10.0 [04 – 16]
Neck pain due to smartphone use	
Never	140 (34.7)
Sometimes	204 (50.6)
Often	59 (14.6)

SD: Standard deviation; Min : Minimum; Max : Maximum.

Table 2 presents the results of the multinomial logistic regression analysis comparing absent and mild, moderate/severe grinding activity. The final adjusted model shows that adolescents who sometimes (OR = 2.102; 95%CI: 1.019–4.339) and often (OR = 4.899; 95%CI: 2.082–11.528) experienced smartphone-induced neck pain were more likely to report mild grinding activity. Adolescents experiencing nightmares at least once a month (OR = 3.402; 95%CI: 1.315–8.802) and sometimes reporting smartphone-induced neck pain (OR = 3.697; 95%CI: 1.103–12.388) were also more likely to report moderate/severe grinding activity.

**Table 2:** Multinomial analysis of the comparison between possible sleep bruxism – grinding activity and sociodemographic characteristics, sleep features, neck pain, and smartphone use among Brazilian adolescents.

Variables	Sleep bruxism – Grinding activity							
	Mild				Moderate/severe			
	OR unadjusted	p-value	OR adjusted	p-value	OR unadjusted	p-value	OR adjusted	p-value
	(95% CI)		(95% CI)		(95%CI)		(95%CI)	
Adolescent’s age	0.953 (0.804–1.128)	0.573	0.926 (0.771–1.113)	0.414	0.812 (0.617–1.070)		0.793 (0.595–1.057)	0.114
Adolescent’s sex								
Male	1		1		1		1	
Female	1.541 (0.880–2.699)	0.130	1.535 (0.846–2.783)	0.158	1.402 (0.601–3.269)		1.552 (0.628–3.834)	0.341
Family income								
≤ 3 BMWs	1				1			
> 3 BMWs	1.981 (1.150–3.411)	**0.014**			1.049 (0.44–2.475)			
Snores during sleep								
No	1		1		1		1	
Sometimes	1.656 (0.949–2.888)	0.076	1.530 (0.852–2.745)	0.154	2.376 (1.027–5.498)		2.181 (0.909–5.232)	0.081
Often	1.147 (0.315–4.175)	0.835	1.146 (0.291–4.504)	0.846	1.182 (0.143–9.781)		1.125 (0.121–10.420)	0.918
Drools on the pillow during sleep								
No	1				1			
Sometimes	2.245 (1.241–4.061)	**0.008**			3.235 (1.208–8.663)			
Often	3.022 (1.280–7.135)	**0.012**			5.288 (1.505–18.585)			
Self-reported hours of sleep	1.032 (0.859–1.241)	0.734			1.135 (0.914–1.561)			
Self-reported sleep quality								
Good	1		1		1		1	
Poor/fairly good	2.030 (1.180–3.493)	**0.011**	1.590 (0.879–2.876)	0.125	2.056 (0.905–4.669)		1.513 (0.620–3.692)	0.363
Has nightmares								
Never/ rarely	1		1		1		1	
At least once a month	1.138 (0.519–2.492)	0.747	0.899 (0.396–2.039)	0.799	4.025 (1.621–9.994)		3.402 (1.315–8.802)	**0.012**
At least once a week	2.717 (1.195–6.180)	**0.017**	2.126 (0.843–5.360)	0.110	2.885 (0.759–10.966)		2.183 (0.519–9.189)	0.287
Neck pain due to smartphone use								
Never	1		1		1		1	
Sometimes	2.266 (1.129–4.550)	**0.021**	2.102 (1.019–4.339)	**0.044**	1.554 (0.567–4.259)		1.321 (0.454–3.840)	0.610
Often	5.382 (2.362–12.262)	**<0.001**	4.899 (2.082–11.528)	**< 0.001**	4.186 (1.319–13.284)		3.697 (1.103–12.388)	**0.034**
Uses the smartphone in bed before bedtime								
No	1				1			
Yes	0.859 (0.406–1.815)	0.690			1.796 (0.408–7.896)			
Uses smartphone to play games before bedtime								
No	1				1			
Yes	0.752 (0.411–1.377)	0.356			1.152 (0.417–3.181)			
Uses smartphone upon waking up, before any other activity								
No	1				1			
Sometimes	0.972 (0.453–2.086)	0.942			1.050 (0.321–3.438)			
Often	1.140 (0.525–2.475)	0.740						
Takes the smartphone to the bedroom when going to sleep								
No	1				1			
Sometimes	0.734 (0.233–2.312)	0.597			0.489 (0.065–3.698)			
Often	0.789 (0.305–2.037)	0.624			0.994 (0.219–4.508)			
Uses the smartphone with lights off after going to sleep								
No	1				1			
Sometimes	0.763 (0.337–1.726)	0.516			2.154 (0.440–10.540)			
Often	0.850 (0.411–1.757)	0.660			2.124 (0.470–9.608)			
Uses the smartphone when waking up in the middle of the night								
No	1				1			
Sometimes	0.935 (0.528–1.658)	0.819			0.757 (0.308–1.862)			
Often	1.243 (0.500–3.087)	0.639			1.294 (0.348–4.803)			
Hours the adolescent thinks they spend on the smartphone per day	1.010 (0.962–1.060)	0.696			1.038 (0.966–1.114)			
Thinks that spends time that should be spent sleeping instead of using the smartphone								
No	1				1			
Yes	1.000 (0.581–1.719)	0.999			1.263 (0.541–2.946)			
Age when first started using the smartphone	0.967 (0.856–1.093)	0.593			0.915 (0.762–1.099)			

OR: odds ratio; CI: confidence interval); P: probability value); BMW: Brazilian minimum wage; BMW = USD$ 283.77Level of significance p < 0.05. Values in bold indicate statistical significance. Category of reference = Absence of sleep bruxism – grinding activity.

Unadjusted and adjusted models for absent and mild, moderate/severe bracing activity are presented in Table 3. The adjusted multinomial model indicates that adolescents who sometimes drooled on the pillow (OR = 3.105; 95%CI: 1.316–7.329) reported having poor/fairly good sleep quality (OR = 2.717; 95%CI: 1.279–5.770) and those who often experienced smartphone-induced neck pain (OR = 3.227; 95%CI: 1.121–9.285) had a higher prevalence of mild bracing activity.

**Table 3:** Multinomial analysis of the comparison between possible sleep bruxism – bracing activity and sociodemographic characteristics, sleep features, neck pain, and smartphone use among Brazilian adolescents.

Variables	Sleep bruxism – Bracing activity							
	Mild				Moderate/severe			
	OR unadjusted	p-value	OR adjusted	p-value	OR unadjusted	p-value	OR adjusted	p-value
	(95%CI)		(95%CI)		(95%CI)		(95%CI)	
Adolescent’s age	1.025 (0.842–1.249)	0.802			1.087 (0.688–1.717)	0.721		
Adolescent’s sex								
Male	1				1			
Female	1.412 (0.728–2.735)	0.307			1.891 (0.362–9.877)	0.450		
Family income								
≤ 3 BMWs	1				1			
> 3 BMWs	1.195 (0.625–2.286)	0.589			1.594 (0.317–8.014)	0.571		
Snores during sleep								
No	1		1		1		1	
Sometimes	1.871 (0.953–3.673)	0.069	1.454 (0.675–3.131)	0.339	1.403 (0.309–6.375)	0.661	00.981 (00.163–50.923)	0.983
Often	3.046 (0.919–10.096)	0.069	2.498 (0.643–9.709)	0.186	-	-	-	-
Drools on the pillow during sleep								
No	1		1		1		1	
Sometimes	2.794 (1.313–5.899)	**0.007**	3.105 (1.316–7.329)	**0.010**	1.707 (0.376–7.755)	0.488	10.260 (00.227–70.001)	0.792
Often	3.332 (1.206–9.204)	**0.020**	2.785 (0.875–8.864)	0.083	-	-	-	-
Self-reported hours of sleep	1.074 (0.867–1.331)	0.513			1.113 (0.676–1.833)	0.673		
Self-reported sleep quality								
Good	1		1		1		1	
Poor	3.028 (1.587–5.778)	**0.001**	2.717 (1.279–5.770)	**0.009**	2.907 (0.640–13.208)	0.167	20.000 (00.374–100.696)	0.418
Has nightmares								
Never/ rarely	1		1		1		1	
At least once a month	1.257 (0.523–3.019)	0.609	1.004 (0.383–2.630)	0.993	1.302 (0.143–11.879)	0.815	00.762 (00.077–70.552)	0.816
At least once a week	2.776 (1.101–6.999)	**0.030**	2.104 (0.681–6.501)	0.196	5.750 (1.001–33.019)	0.050	60.062 (00.697–520.747)	0.103
Neck pain due to smartphone use								
Never	1		1		1		1	
Sometimes	1.433 (0.649–3.165)	0.373	1.182 (0.464–3.009)	0.725	2.867 (0.317–25.948)	0.349	30.697 (00.316–430.178)	0.297
Often	3.811 (1.561–9.305)	**0.003**	3.227 (1.121–9.285)	**0.030**	5.864 (0.519–66.254)	0.153	110.372 (00.724–1780.709)	0.084
Uses the smartphone in bed before bedtime								
No	1				1			
Yes	1.582 (0.541–4.623)	0.402			0.974 (0.115–8.261)	0.980		
Uses smartphone to play games before bedtime								
No	1				1			
Yes	1.345 (0.600–3.013)	0.471			0.769 (0.146–4.034)	0.756		
Uses smartphone upon waking up, before any other activity								
No	1				1			
Sometimes	2.444 (0.697–8.577)	0.163			1.222 (0.125–11.964)	0.863		
Often	3.872 (1.118–13.415)	**0.033**			1.584 (0.162–15.529)	0.693		
Takes the smartphone to the bedroom when going to sleep								
No	1		1		-	-	-	-
Sometimes	3.462 (0.398–30.141)	0.261	3.355 (0.352–31.984)	0.293	-	-	-	-
Often	3.985 (0.527–30.116)	0.180	2.768 (0.321–23.896)	0.355	-	-	-	-
Uses the smartphone with lights off after going to sleep								
No	-	-			-	-		
Sometimes	-	-			-	-		
Often	-	-			-	-		
Uses the smartphone when waking up in the middle of the night								
No	1		1		1		1	
Sometimes	1.100 (0.550–2.201)	0.788	0.666 (0.291–1.526)	0.337	1.031 (0.227–4.683)	0.968	00.885 (00.160–40.908)	0.889
Often	2.182 (0.850–5.602)	0.105	0.665 (0.188–2.357)	0.528	-	-	-	-
Hours the adolescent thinks they spend on the smartphone per day	1.056 (1.002–1.114)	**0.042**	1.047 (0.982–1.117)	0.157	0.904 (0.747–1.093)	0.296	00.851 (00.655–10.105)	0.225
Thinks that spends time that should be spent sleeping instead of using the smartphone								
No	1				1			
Yes	1.495 (0.764–2.298)	0.240			0.541 (0.119–2.455)	0.426		
Age when first started using the smartphone	0.990 (0.857–1.145)	0.897	0.997 (0.841–1.181)	0.969	1.283 (0.884–1.861)	0.189	10.338 (00.837–20.139)	0.223

OR: odds ratio; CI: confidence interval); P: probability value); BMW: Brazilian minimum wage; BMW = USD$ 283.77.Level of significance p < 0.05. Values in bold indicate statistical significance. Category of reference = Absence of sleep bruxism – bracing activity.

Table 4 presents the results of the multinomial analysis comparing absent and mild, moderate/severe thrusting activity. The adjusted model shows that adolescents experiencing nightmares at least once a month (OR = 2.540; 95%CI: 1.127–5.725) and once a week (OR = 3.209; 95%CI: 1.202–8.565) were more likely to report mild thrusting activity.

**Table 4:** Multinomial analysis of the comparison between possible sleep bruxism – thrusting activity and sociodemographic characteristics, sleep features, neck pain, and smartphone use among adolescents from Belo Horizonte, Brazil.

Variables	Sleep bruxism – Thrusting activity							
	Mild				Moderate/severe			
	OR non-adjusted	p-value	OR adjusted	p-value	OR non-adjusted	p-value	OR adjusted	p-value
	(95%CI)		(95%CI)		(95%CI)		(95%CI)	
Adolescent’s age	0.926 (0.757–1.134)	0.458			1.168 (0.847–1.611)	0.344		
Adolescent’s sex								
Male	1		1		1		1	
Female	1.708 (0.875–3.334)	0.117	1.464 (0.717–2.990)	0.295	4.781 (1.054–21.685)	**0.043**	4.347 (0.912–20.729)	0.065
Family income								
≤ 3 BMWs	1				1			
> 3 BMWs	0.920 (0.478–1.773)	0.804			1.331 (0.438–4.048)	0.614		
Snores during sleep								
No	1				1			
Sometimes	1.487 (0.766–2.886)	0.241			1.010 (0.331–3.084)	0.987		
Often	2.533 (0.773–8.304)	0.125			-	-		
Drools on the pillow during sleep								
No	1		1		1		1	
Sometimes	1.204 (0.593–2.445)	0.607	1.020 (0.486–2.140)	0.958	2.408 (0.711–8.163)	0.158	2.024 (0.564–7.262)	0.279
Often	3.018 (1.231–7.395)	**0.016**	2.503 (0.975–6.428)	0.057	2.850 (0.500–16.256)	0.238	1.888 (0.307–11.592)	0.493
Self-reported hours of sleep	0.913 (0.731–1.141)	0.425			1.110 (0.778–1.583)	0.564		
Self-reported sleep quality								
Good	1		1		1		1	
Reasonable/ Bad	2.371 (1.258–4.469)	**0.008**	1.729 (0.853–3.502)	0.128	2.887 (0.978–8.523)	0.055	1.795 (0.553–5.827)	0.330
Has nightmares								
Never/ rarely	1		1		1		1	
At least once a month	2.699 (1.242–5.864)	**0.012**	2.540 (1.127–5.725)	**0.025**	3.833 (1.201–12.239)	**0.023**	3.216 (0.929–11.141)	0.065
At least once a week	3.680 (1.498–9.040)	**0.004**	3.209 (1.202–8.565)	**0.020**	1.438 (0.172–11.979)	0.737	1.015 (0.108–9.529)	0.990
Text neck								
Never	1		1		1		1	
Sometimes	2.222 (1.007–4.905)	**0.048**	1.749 (0.745–4.104)	0.199	6.923 (0.866–55.338)	0.068	4.428 (0.516–37.995)	0.175
Often	2.826 (1.057–7.554)	**0.038**	1.813 (0.623–5.281)	0.275	11.304 (1.232–103.766)	**0.032**	5.244 (0.512–53.693)	0.163
Uses the smartphone in bed before bedtime								
No	1				-	-		
Yes	0.839 (0.354–1.992)	0.691			-	-		
Uses smartphone to play games before bedtime								
No	1				1			
Yes	1.043 (0.494–2.203)	0.912			1.841 (0.404–8.396)	0.431		
Uses smartphone upon waking up, before any other activity								
No	1				1			
Sometimes	1.163 (0.441–3.065)	0.761			0.969 (0.183–5.125)	0.970		
Often	1.680 (0.642–4.397)	0.290			1.764 (0.356–8.746)	0.487		
Takes the smartphone to the bedroom when going to sleep								
No	-	-			-	-		
Sometimes	-	-			-	-		
Often	-	-			-	-		
Uses the smartphone with lights off after going to sleep								
No	1		1		1		1	
Sometimes	2.367 (0.755–7.423)	0.139	2.043 (0.601–6.940)	0.252	1.184 (0.105–13.342)	0.891	1.125 (0.085–14.853)	0.929
Often	1.841 (0.614–5.524)	0.276	1.226 (0.366–4.106)	0.741	3.376 (0.427–26.702)	0.249	1.652 (0.177–15.391)	0.660
Uses the smartphone when waking up in the middle of the night								
No	1				1			
Sometimes	0.688 (0.344–1.395)	0.300			1.324 (0.418–4.194)	0.634		
Often	1.490 (0.563–3.943)	0.422			2.069 (0.398–10.748)	0.387		
Hours the adolescent thinks they spend on the smartphone per day	1.027 (0.971–1.085)	0.352			1.071 (0.984–1.164)	0.112		
Thinks that spends time that should be spent sleeping instead of using the smartphone								
No	1		1		1		1	
Yes	1.856 (0.939–3.670)	0.075	1.515 (0.699–3.285)	0.292	4.670 (1.030–21.182)	**0.046**	2.542 (0.496–13.020)	0.263
Age when first started using the smartphone	0.963 (1.110–0.835)	0.601			1.240 (0.952–1.614)	0.110		

OR: odds ratio; CI: confidence interval); P: probability value); BMW: Brazilian minimum wage; BMW = USD$ 283.77. Level of significance p < 0.05. Values in bold indicate statistical significance. Category of reference = Absence of sleep bruxism – bracing activity.

## Discussion

The present study demonstrated an association of PSB activities with smartphone-induced neck pain, poor/fairly good sleep quality, history of nightmares, and drooling on the pillow while sleeping. Bruxism activity might be a risk factor for myofascial neck pain.^10,14,19,20^This hypothesis had been tested in a study evaluating jaw muscles and neck muscle activity during several bite tasks, simulating bruxism activity.^19^A possible explanation for the association between PSB and neck pain could be related to head posture. Previous studies with children and young adults have found an association between SB and forward head posture,^11,12^ possibly as a way to improve respiratory muscle function.^12,21^The excessive use of smartphones in patients with SB, who may already have altered head posture,^12,21^could exacerbate musculoskeletal disorders and worsen neck pain. This could explain the association between neck pain and bracing and grinding activities. In this study, neck pain was specifically linked to smartphone use, as indicated in the questionnaire. Smartphone-induced neck pain can be one of several complications of the text neck syndrome.^22^ This syndrome can affect the eyes, the heart, the lungs, the head, and psychological well-being.^22^ Further research is needed to investigate different SB-related activities, neck pain, text neck syndrome, and smartphone use.

In our study sample, all participants used smartphones, and a high percentage reported using them in bed before bedtime and immediately upon waking up in the morning. These findings are consistent with previous research.^23^ A Brazilian study examining electronic device exposure and sleep features found that 89.5% of adolescents used smartphones on weekdays, while 90.6% used them on weekends.^23^Late-night smartphone use was associated with poor sleep quality and duration.^23^ Other studies have shown a high percentage of children and adolescents sleeping less than the recommended eight hours per night, as recommended by the American Academy of Sleep Medicine.^24^Increased social media use may be the main contributing factor to sleep problems in this age group, with more than two-thirds of adolescents using smartphones before bedtime.^25^

Although smartphone use was not directly associated with PSB activities, the device could indirectly affect SB activities. Musculoskeletal changes associated with excessive smartphone use are still emerging in the literature, but many experts acknowledge this association.^5^ During smartphone use, increased muscle activity, head angulation, and forward head posture may contribute to musculoskeletal pain.^8^ When using a smartphone, young individuals often flex their heads at an angle of 33º to 45º, significantly increasing the load on the cervical spine.^26^ While in a neutral position, the head weighs 4.5 kg to 5.4 kg; at an angle of 30º, it weighs 18 kg; and at 45º, it weighs 22 kg, approximately.^27^ This is of particular concern among adolescents, as their spines are still developing and are more susceptible to ligament contractures and accelerated cervical disc degeneration.^5^

Another factor associated with bracing was drooling on the pillow during sleep. Previous studies that had not considered different SB activities have also reported an association between SB and drooling.^4^ Drooling on the pillow can indicate mouth breathing, snoring, sleeping with the mouth open, and frequent nasal obstruction in children.^28^ According to the current bruxism consensus, “bracing” refers to forcefully maintaining a specific mandibular position,^1^ which may help airflow and salivary flow during sleep, particularly in adolescents with mouth or sleep-disordered breathing such as obstructive sleep apnea.^2^ In this case, bracing would work as a protective factor for respiratory obstruction. However, the exact role of non-tooth-contact SB activities and their protective effects remains unclear and warrants further investigation.^1^

Bracing was also associated with poor/fairly good sleep quality. This finding is in line with previous studies involving children.^29^ Low-intensity contractions of craniofacial muscles, part of SB activity, can lead to muscle pain, fatigue, and increased stress levels, potentially affecting sleep quality.^30^Forcefully maintaining a specific mandibular position during sleep could also interfere with sleep quality and exacerbate respiratory problems frequently associated with SB.^1,2^

Reports of nightmares were associated with mild thrusting and moderate/severe grinding. A previous study with adolescents has also found an association between self-reported nightmares and SB.^31^ Nightmares are disturbing dreams that evoke negative emotions and may lead to arousals,^32^ contributing to biological factors associated with SB.^2^ Adolescents experiencing nightmares may have sleep arousals, leading to increased thrusting and grinding activities during sleep.

Some limitations of this research should be addressed. Polysomnography recording, considered the gold standard for assessing SB, has limited application in epidemiological research because of its high cost.^1,4^ While using a frequency-based severity grading scale is a consensus recommendation for investigating PSB activities,^1^ its application in investigations involving adolescents can be challenging given the complexity of the questions. To minimize potential bias, we formulated all questions using clear and age-appropriate language for the sample’s age group and tested them in a pilot study, in which adolescents reported no difficulties with PSB questions or other items. Additionally, evidence is definitely inconclusive as to whether dichotomized (yes or no) or frequency-based (never, sometimes, or often) PSB assessments are more reliable. Frequency-based assessment may lead to the dispersion of individuals across categories, potentially explaining the low prevalence of moderate/severe PSB and the broad confidence intervals observed in regression models. The low prevalence of moderate/severe PSB may also be characteristic of the age group studied, as reported in studies involving children.^14^ Further research is needed to compare the reliability of assessment methods and to focus on the prevalence of different PSB activities involving larger samples of adolescents.

Neck pain and smartphone use assessments were based on adolescents’ self-reports. At the time of data collection, no validated instrument was available in Brazilian Portuguese for this age group. The question evaluating neck pain was specific to smartphone use; however, it is possible that other factors than smartphone use could also trigger neck pain. Future studies employing alternative instruments and assessment methods are encouraged to validate and build upon the current findings. Additionally, it is important to note that self-reported instruments rely on an individual’s ability to recall past events, which may introduce recall bias.^4^

The use of smartphones has promoted changes in society, transforming communication across all age groups. This has contributed to emerging health issues, such as neck pain and insufficient sleep.^5,24^ The impact of excessive smartphone use on oral health is still under investigation, but its effects on sleep and musculoskeletal structures have been confirmed by recent studies.^5,22^ SB is a complex behavior influenced by several etiologic factors, including sleep features. Understanding its relation with other health issues, such as musculoskeletal conditions, could assist clinicians in effectively managing and controlling SB.^2^ Therefore, clinicians and researchers are encouraged to incorporate assessments of smartphone use and sleep features into routine anamnesis. Such assessments can help establish links between these factors and oral conditions such as SB, promoting a comprehensive approach from diagnosis to treatment.

## Conclusion

Self-reported neck pain caused by smartphone use, history of nightmares, poor/fairly good sleep quality, and drooling on the pillow were associated with a higher prevalence of PSB activities among Brazilian adolescents.
